# The Rationale for Photobiomodulation Therapy of Vaginal Tissue for Treatment of Genitourinary Syndrome of Menopause: An Analysis of Its Mechanism of Action, and Current Clinical Outcomes

**DOI:** 10.1089/photob.2019.4618

**Published:** 2019-07-01

**Authors:** Raymond J. Lanzafame, Sarah de la Torre, Gustavo H. Leibaschoff

**Affiliations:** ^1^ Raymond J. Lanzafame, MD PLLC, Rochester, New York.; ^2^ Seattle OB/GYN Group, Seattle, Washington.; ^3^ International Consultants in Aesthetic Medicine, Dallas, Texas.

**Keywords:** womens' health, genitourinary syndrome of menopause, vagina, tissue regeneration and healing, photobiomodulation, collagen, stress urinary incontinence

## Abstract

***Objective:*** Light, particularly in the visible to far-infrared spectrum, has been applied to the female genital tract with lasers and other devices for nearly 50 years. These have included procedures on both normal and neoplastic tissues, management of condylomata, endometriosis, and menometrorrhagia, and, more recently, a number of fractional laser devices have been applied for the management of genitourinary syndrome of menopause (GSM) and stress urinary incontinence (SUI), and to achieve so-called vaginal rejuvenation. Photobiomodulation therapy (PBMT) has been proposed as an alternative for use in managing GSM and SUI.

***Methods:*** This article reviews the biological basis, symptoms, and management of GSM, and investigates the current status and rationale for the use of PBMT.

***Results and conclusions:*** Based on the preliminary evidence available, PBMT is safe and appears to be efficacious in treating GSM.

## Introduction

Both the public and the medical community at large are becoming more aware and vocal regarding women's health and related issues. This has included frank discussions of self-awareness and wellness, intimacy, body image ideals, aging and antiaging strategies, as well as cancer prevention and treatment, among other health issues.

Light, particularly in the visible to far-infrared spectrum, has been applied to the female genital tract with lasers and other devices to diagnose and/or treat various diseases for nearly 50 years. These have included procedures on both normal and neoplastic tissues, ablation and excision of condylomata, endometriosis lesion excision and vaporization, and the management of menometrorrhagia and other conditions. More recently, a number of fractional laser devices have been applied for the management of genitourinary syndrome of menopause (GSM) and stress urinary incontinence (SUI), and to achieve so-called vaginal rejuvenation. The Food and Drug Administration (FDA) has noted that despite the use of various devices for GSM and SUI, it has not approved any devices specifically for these indications and cites adverse events and poor outcomes after use of these technologies.^1,2^

Photobiomodulation therapy (PBMT) has been proposed as an alternative for use in managing GSM and SUI. This article reviews the biological basis, symptoms, and management of GSM, and investigates the current status and rationale for the use of PBMT to treat these conditions. The characteristics of the devices in current use and available clinical and histologic data are presented.

## Genitourinary Syndrome of Menopause

The vaginal epithelium undergoes changes in response to the level of circulating estrogens during the female reproductive years. Circulating estrogen levels show an affected reduction when menopause occurs. The lack of estrogen induces several modifications of the genital tissues that correlate with the beginning of systematic negative effects. Significant cytological transformations follow estrogen reduction, including the proliferation of connective tissue, the fragmentation of elastin, and collagen hyalinization. These changes may result in a condition described as vulvovaginal atrophy (VVA).^3^

Estrogen receptors have been identified in the pelvic floor musculature and tissues and these are susceptible to estrogen deprivation as occurs in menopause.^3–7^ Vaginal wall connective tissue components, including collagen, elastin, and smooth muscle, all degenerate as a result of estrogen deprivation. The vaginal epithelium subsequently becomes less cellular and thinner, and glycogen production, which is responsible for vaginal secretion, gradually declines and ultimately comes to a complete halt. Blood flow to the vagina is also reduced, which is associated with decreased fluid secretion during sexual arousal. These changes produce a variety of symptoms, and most notably, vaginal dryness, and the resultant decrease in natural lubrication increases susceptibility to trauma and pain during intercourse.^3–7^

Up to two-thirds of menopausal women have physical evidence of VVA and ∼50% of postmenopausal women have VVA symptoms. The major findings and symptoms of VVA are decreased vaginal lubrication, leading to vaginal dryness, followed by other vaginal and urinary symptoms, such as burning, itching, bleeding, leucorrhea, dyspareunia, and dysuria.^3,6–10^ These symptoms usually appear ∼2–4 years after the onset of menopause.

Symptoms of VVA are not limited to sexually active women with their severity ranging from mild to debilitating, and they can negatively affect patients' quality of life (QoL).^3,6,7^ VVA symptoms have an adverse emotional and physical impact on patients and their partners by contributing to unsatisfactory sexual relationships.^10–12^

The term GSM emerged following a consensus conference held in May 2013 in consideration of the fact that VVA is also associated with a wide range of urogynecological symptoms, including urinary incontinence.^12,13^ Menopausal women can experience a decrease in both the diameter and quantity of periurethral striated muscles. These changes may be responsible for the observed functional effects on intrinsic and extrinsic continence mechanisms. In addition, collagen I and III content and quality at the level of the endopelvic fascia are affected and have been shown to have a close relationship with pelvic floor dysfunction, including SUI, which is the most common of the incontinence dysfunctions.^14^

Decreased vaginal tissue and muscular function is basically a result of damage to the vagina at a nerve, muscular, cellular, and connective tissue level caused primarily by tears and stress on the pelvic floor during pregnancy and vaginal delivery, as well as the breakdown of vaginal tissue and decreased blood flow of menopause due to estrogen deprivation.^14–18^ An understanding of the determinants of vaginal tissue strength and integrity is important in designing appropriate management strategies to treat GSM.

The connective tissue of the vagina is composed of fibroblasts, and smooth muscle cells, surrounded by an extracellular matrix (ECM).^14^ Although fibroblasts are the main cells responsible for the synthesis and secretion of fibrillar collagen and elastin and less abundant nonfibrillar components, smooth muscle cells can also synthesize these molecules. Collagen and elastin are fundamental components that control the biomechanical properties of the vaginal tissue. The ECM is constantly remodeling, and its homeostasis depends on the balance between synthesis and degradation by matrix metalloproteinases (MMPs), which are further controlled by activators and tissue inhibitors of MMPs. Both processes are modulated by soluble biological mediators, including growth factors and their receptors, as well as by chemical and mechanical signaling from the ECM, which is recognized by transmembrane receptors and integrins.^19^

Fibrillar collagens are the principal determinants of vaginal tissue strength. Collagen I, III, and V are the main collagen subtypes present in the vagina. Collagen I is abundant in skin, ligament, tendon, and bone. It forms large and strong fibers that are responsible for the mechanical resistance of tissue. Collagen III forms smaller fibers with lower tensile strength and is present in mobile organs and tissues that are cyclically stretched such as blood vessels.^19^ Collagen V forms small fibers of low tensile strength. Collagens I, III, and V copolymerize to form hybrid fibrils. Collagen V forms the fibril core that is surrounded by copolymers of collagen I and III. The proportion of each subtype of collagen determines the fiber size and has an impact on the biomechanical strength of the tissue.^19^ The collagen fibers are further covered by collagens XII and XIV and small leucine-rich proteoglycans such as decorin, which also participate in the control of fibrinogenesis. Elastic fibers are key architectural elements of connective tissues that are subject to mechanical tension and expansile forces. They provide extensibility and recoil in elastic tissues and are important in maintaining vaginal structural integrity against mechanical strain.^18^

GSM is unlikely to improve over time without treatment, and the signs and symptoms of GSM tend to reappear if treatment is discontinued.^20,21^ The main therapeutic objectives in managing GSM are to relieve genital symptoms and to attempt to restore the vaginal environment to a healthy condition.^6^ To-date, the most common treatments for GSM include interactive therapy using pelvic floor exercises, physical therapy, bladder training and timed canceling, pharmacologic therapy, and/or surgical intervention for those patients suffering from incontinence-related issues. Vaginal estrogens are most commonly prescribed for women suffering from VVA symptoms. Unfortunately, pelvic floor exercises have limited long-term success due to compliance issues^22,23^ and there are a significant number of postmenopausal women who are not candidates for use of estrogen-based creams, given a concern for increased risk as a result of previous breast or other cancers. The optimal approach for the relief of GSM symptoms needs to take into consideration patient preferences and willingness to comply with treatment regimens, in addition to being mindful of contraindications to therapy.

## Current Nonpharmacologic Therapies

Various nonpharmacologic modalities have been used in the vagina and on the vulva both for therapeutic and recreational purposes. We discuss these in the context of their current applications and mechanism of action.

### Vibration

Vibrators and self-stimulators have been applied to the vulva and vagina in a variety of forms over the past 100 years for sexual play, self-stimulation, as well as for therapeutic purposes. Vibration stimulates the vulvar and vaginal tissues, which may facilitate natural lubrication and help maintain function.^24^

### Thermal energy

Thermal energy has been applied to the vulva and vagina in a number of forms and in a number of devices for both nontherapeutic and therapeutic reasons. Examples of some of these include the following: bathing in warm water, warm water douches, warmed intravaginal lubricants, exposure to heat lamps, use of electric stimulation incontinence devices, use of electrical vibrators, intravaginal ultrasound, and various radiofrequency (RF) and laser devices.^24–30^

RF, CO_2_, and Er:YAG devices have been approved for use in the vulva and lower female genital tract.^24–30^ Each of these modalities produce local effects in the vaginal mucosa and subjacent tissues in part or totally via a thermal heating mechanism and, in part, by induction of heat shock proteins (HSP) and induction of a cascade of local effects.^24–30^

In RF-based devices, RF energy is directed to specific tissue depths to achieve and maintain temperatures in the range of 40°C–45°C.^24,26–29^ The delivered RF energy does not result in tissue ablation or removal as is the case with the laser technologies.

CO_2_ and Er:YAG devices exert their effects via specifically configured beams of light energy in the far-infrared and midinfrared spectrum, respectively. Their beam temperatures are in the range of 1500°C, although the tissue temperature at the site of impact (zone of ablation) reaches 100°C due to the boiling point of water, and surrounding skin temperatures reach 120°C–200°C in the case of the CO_2_ laser.^24–29^ Recently, Er:YAG laser technology producing minimal ablation using 300 μs superpulsed mode with 50 ms intrapulse intervals and a pulse duration of 250 ms has been developed.^25,27^ This technology raises the vaginal mucosal temperature to 60°C–65°C.^25,27^

HSP induction via thermal stress in normal tissue has not been associated with genotoxic outcomes, nor with potentiation of genotoxicity in the presence of adjuvants. Similarly, there have been no reports of abnormal cytologic or histologic changes being observed in patients who have undergone various vaginal or lower genital tract procedures with these technologies. Of particular note, CO_2_, Nd:YAG, and various other laser devices have been used for nearly five decades.^27^

Studies have demonstrated that the controlled deposition and use of thermal energy on the vaginal wall are capable of stimulating the proliferation of glycogen-enriched epithelium, neovascularization, collagen formation in the lamina propria, improvements in natural lubrication, and urinary control.^24–29^

### Photonic energy

Light, particularly in the visible to far-infrared spectrum, has been applied to the female genital tract with lasers and other devices for nearly five decades.^27^ A variety of delivery devices, including free beam applications and colposcopic, microscopic, fiberoptic, and other contact probes, have been used to incise, coagulate, vaporize, and ablate tissues. These have included procedures on both normal and neoplastic tissues, and management of condylomata, endometriosis, menometrorrhagia, and other conditions. More recently, a number of fractional laser devices have been applied for the management of GSM and SUI, and to achieve so-called vaginal rejuvenation.^24–39^ However, it should be noted that to-date, the FDA has not specifically approved any devices for these indications.^1,2^ Several devices emitting in the red range of the spectrum have been used in conjunction with porphyrins and other agents for photodynamic therapy to treat various malignant or premalignant lesions.^27^

### Photobiomodulation therapy

PBMT is being used clinically and experimentally for a variety of processes and conditions.^39–95^ Light sources, including lasers, light-emitting diodes (LEDs), supraluminous diodes (SLDs), and other noncoherent sources, are used both clinically and experimentally. PBMT with red and near-infrared radiation (NIR) light has been applied clinically for a diverse array of indications, including facial rejuvenation and treatment of skin laxity, wound healing, pain management, reduction of inflammation, stimulation of hair regrowth, and treatment of acne.

Vaginal tissue, such as skin, can experience laxity as women age or go through childbirth. Microcirculation and blood flow in vaginal and urethral tissues decrease with menopause, and the decline in estrogen negatively impacting vaginal hydration and bladder function. The current rationale for treating the vaginal tissue with PBMT is to stimulate synthesis of collagen and elastin in the vaginal tissue and supporting urethrovaginal sphincter and urethra, as well as promoting vasodilation in the vaginal and urethral submucosa.

### Photobiomodulation mechanism of action

The widely accepted theory is that photobiomodulation, particularly at wavelengths in the red range, activates cytochrome c oxidase and increases mitochondrial electron transport, leading to an increase in adenosine triphosphate (ATP).^47,49–58,60,61^ Photoacceptors in the tissue cells' mitochondria absorb red and NIR light, inducing a cascade of events resulting in the production of reactive oxygen species (ROS), nitric oxide (NO), and reactive nitrogen species.

Healing is affected by upregulation of specific substrates and inhibition of others. Collagen production is stimulated, various cytokines are upregulated [e.g., epidermal growth factor, transforming growth factor beta, and fibroblast growth factor (FGF)], and inflammatory cytokines [e.g., interleukin (IL)-6, IL-8, and IL-1] are downregulated by photobiomodulation. Upregulation of cytochromes, other transport and energy compounds [e.g., nicotinamide adenine dinucleotide hydrogen (NADH), ATP, and adenosine diphosphate (ADP)], enhances the activities of various cellular components in the local wound milieu.^39–48^

Avci et al. reviewed the use of PBMT for the treatment of hair loss.^60^ They reported that PBMT acts on the mitochondria and may alter cell metabolism through photodissociation of inhibitory NO from cytochrome c oxidase, specifically Unit IV in the respiratory chain of mitochondria, causing increased ATP production, modulation of ROS, and induction of transcription factors, such as nuclear factor kappa B, and hypoxia-inducible factor-1. These transcription factors in return cause protein synthesis that triggers further effects down-stream, such as increased cell proliferation and migration, alteration in the levels of cytokines, growth factors, and inflammatory mediators, and increased tissue oxygenation. They note further that NO is known to be a potent vasodilator via its effect on cyclic guanine monophosphate production and it can be speculated that PBMT may cause photodissociation of NO not only from cytochrome c oxidase but also from intracellular stores such as nitrosylated forms of both hemoglobin and myoglobin leading to vasodilation and increased blood flow.^60^

The interaction of photons with cells is a necessary and essential condition for photobiomodulation to occur. Absorption and transduction of this energy must occur and it is well known that cellular molecules and structures are capable of absorbing this energy at various wavelengths.^45,47,49–58,60–62^ It is also known that transmission of some portion of the incident light occurs depending on the wavelength, the irradiance, the time course of the interaction, and the particular tissue being exposed to the beam. While it is likely that a number of intracellular molecules are capable of transduction of light energy in eukaryotic cells, the cytochrome system is the primary target. Several investigators have documented that photobiomodulation upregulates ATP production via this system.^45,47,49–58,60,61^ However, some cells and tissues remain unresponsive to phototherapy, even when it is provided according to generally accepted and published parameters.^60,61,67^

Lanzafame demonstrated this phenomenon in a simple tissue model using fetal bovine heart endothelial cells (FBHE).^67^ These cells require growth factors for growth and proliferation. They have an absolute dependency on basic FGF (bFGF) for survival in tissue culture.^67^ They demonstrated that 660 nm photoradiation at 2.16 J/cm^2^ significantly increased cell proliferation and bFGF production in fibroblasts, which in turn increased proliferation of FBHE. Photoirradiation of the FBHE alone did not result in changes in cell proliferation.^67^ Both cell lines possess mitochondria and cytochromes, but FBHE still require bFGF and are otherwise “refractory” or “unresponsive” to light exposure.

PBMT has been demonstrated to accelerate wound healing^39–46,48,54,55,74^ and reduce pain and inflammation^63,66,71,73^ and cancer.^75–79^ PBMT augments intracellular metabolism by increasing ATP production, among other metabolic pathways, as well as to induce or reduce production of ROS and other free radicals as the mechanistic basis for the outcomes observed after using PBMT.^43–58,63,64,66,71–73^ The ability of PBMT to downregulate inflammation and modulate inflammatory processes via cellular metabolism and cellular responses induced by changes in ROS, NO, and other inflammatory mediators orchestrated by light exposure might also contribute to its mechanism of action.^52–54,57,58,60–67,71,73,77^

Work from several laboratories has demonstrated that NIR photoirradiation can accelerate wound healing and tensile strength in both normal and impaired tissues and that the effects are not related to alterations in tissue temperature.^48,68,69,74,96^ Tissue tensile strength increases are correlated with increased collagen synthesis and tissue collagen content, demonstrated in various models and with both red and NIR wavelengths.^48,68,69,74,96^

## Safety Considerations Regarding PBMT of Vaginal Tissue

The FDA recently issued a statement indicating that there are no energy-based devices currently marketed in the United States that have been cleared for vaginal cosmetic procedures, including indications for managing GSM, SUI, or other menopause-related conditions.^1,2^ The FDA statement and subsequent releases cite numerous adverse events from use of lasers and RF sources for these indications. These include both specific surgically related complications and thermal injuries causing burns, scarring, and pain. The FDA further notes that the safety and efficacy of the devices used have not been established.^1^

Photobiomodulation devices and therapies exert their effects via nonthermal mechanisms. PBMT has demonstrated a remarkably low incidence of adverse effects, having been used for more than 50 years for diverse medical conditions and in a variety of anatomical sites.^39–45,57–62,65,66,70–73,82,83,86–90,93^ Analysis of nonphotoradiated and photoirradiated tissues has been used to elucidate the tissue response and efficacy of the photobiomodulation effect.^39,58,60–63,65^ Haywood et al. found no detectable free radicals after exposure of human skin biopsy to 694 nm light at 11–14 J/cm^2^ in 0.9 ms pulses using electron spin resonance spectroscopy.^65^

To the best of our knowledge, there are no reports of damage to biological tissues such as sweat glands, blood vessels, skin, muscle, or other adjacent structures by PBMT.^39,55,64–66,75–79^ Similarly, there is no report that these wavelengths induce genotoxic changes, mutagenesis, or cancer, even in patients undergoing chemotherapy and who are receiving PBMT for prevention and management of oral mucositis.^81–92^

To-date, there has been limited use of photobiomodulation devices for PBMT of vaginal tissues. However, the use of PBMT for the management of oral mucositis and other oral conditions^83–92^ supports the hypothesis that use of PBMT for vaginal treatment is reasonable. Thompson demonstrated that vaginal and buccal epithelia are microscopically similar.^84^ They noted that there were no statistically significant differences between the maximum and minimum number of epithelial cell layers. Further, the patterns of surface keratinization and the distribution and appearance of the lipid lamellae in the intercellular spaces are similar. Thompson concluded that based on these findings, the buccal epithelium can be used as a surrogate for the vaginal epithelium.^84^

Neuman and Finklestein evaluated 660 nm PBMT on the nasal clinical symptoms of allergic rhinitis in a double-blinded randomized prospective study.^89^ A statistically significant improvement of symptoms was reported by 72% of the allergic rhinitis patients and objective improvement was endoscopically demonstrated in 70% of the patients treated by narrow-band red light illumination of the nasal mucosa at 660 nm when compared with the placebo group, with marked alleviation of clinical symptoms.^89^

PBMT has also been shown to be an effective treatment for other inflammatory processes in an animal model. Zigmond evaluated the effect of PBMT on mucosal healing in an acute colitis model in mice.^85^ PBMT was applied to the colon utilizing a small-diameter endoscope with an LED-based light source at wavelengths of 440, 660, and 850 nm. PBMT commenced 1 day before induction of colitis and continued during the 6-day induction period, as well as for 3–10 days thereafter. Disease activity was scored endoscopically and by histopathological assessment. A statistically significant improvement in disease severity was observed in all 3 PBMT treatment groups.^85^

## Data on the Use of PBMTs on Vaginal Tissue

Preliminary clinical data concerning the use of devices using photonic sources operating at parameters typically used for PBMT for the treatment of vaginal tissues are discussed below. The characteristics of these devices, their existing pre-clinical and clinical data, and preliminary histologic analysis of tissue response to therapy are presented.

### Device characteristics

Joylux, Inc. (Seattle, Washington) introduced PMBT as a component of devices for the treatment of vaginal tissues for GSM management under the brand names vSculpt and vFit. vSculpt and vSculpt PRO are cleared as Class IIa medical devices for SUI, dryness, and sexual dysfunction outside of the United States. The vFit and vFit PLUS devices are classified as Low Risk General Health and Wellness Devices by the FDA with claims for improvement in general vaginal wellness and sexual function. These devices apply thermal loading and photonic and vibrational energy to the vagina. The total photonic power output of the devices ranges between 1.2 and 1.4 W, with delivered energy densities of 16–24 J/cm^2^, depending on time and model. Each of these modalities is being used for the promotion of vaginal wellness and other therapeutic indications, using a variety of delivery devices and energy sources to achieve their desired effects.^24–29^ Given the fact that the vSculpt device represents a combination product by virtue of use of vibrational, thermal, and photonic energy, it is necessary to consider the nonoptical components in the context of understanding the role of photobiomodulation as applied to vaginal tissues via this particular instrument.

**FIG. 1.**
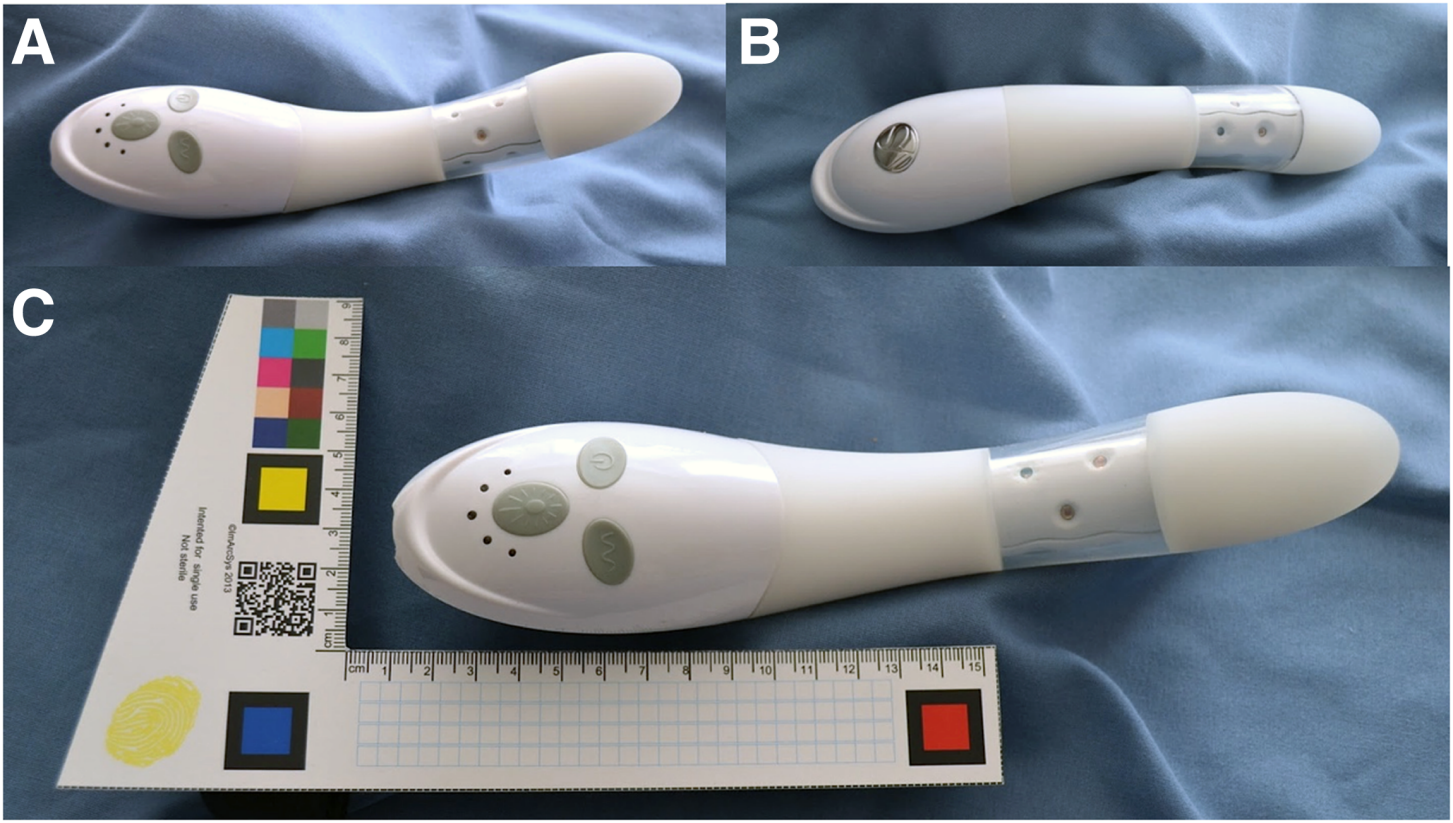
The vSculpt device is demonstrated. The anterior aspect (top) of the device is shown **(A, C)**. The posterior aspect (bottom) of the device is shown **(B)**. The scale of the device is demonstrated **(C)**.

**FIG. 2.**
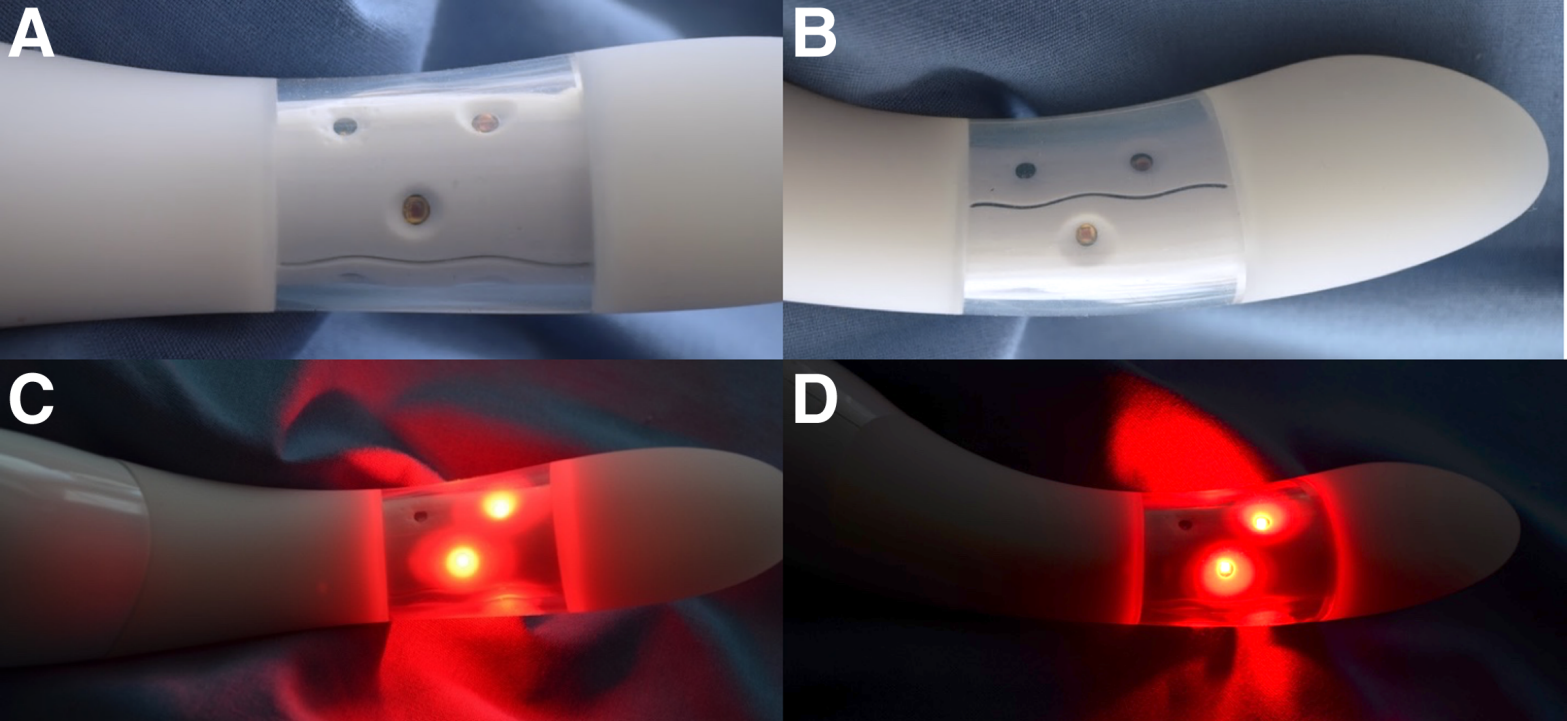
The vSculpt LED source configuration and light distribution are demonstrated. Anterior device views are shown both without **(A)** and with device activation **(C)**. Lateral device views are shown both without **(B)** and with device activation **(D)**. LED, light-emitting diode.

The vSculpt device contains two vibration motors with six vibration mode options. Sonic technology produces vibration in the 75–110 Hz range. The device contains rechargeable lithium ion batteries with a power input of 5 V, and 100 mA located in the external (handle) portion of the device (Fig. 1). These remain external to the vagina during use. Heat is produced by vibration and is measured to be 40°C–42°C at 10 min and 40°C–44°C at 12 min of treatment. Internal thermistors and an aluminum core heat sink are incorporated within the unit to prevent overheating during use. Most of the heat generated occurs in the section of the controls, followed by the area above the LEDs. The remaining heat that is not absorbed by the heat sink dissipates around the LEDs in the middle of the shaft of the device (Figs. 1 and 2), and less being transmited to and dissipated at the tip of the unit.

Two thermal studies were conducted to measure the temperature of vaginal tissue after vSculpt use. The first study was a pilot study using thermography to measure the temperature of the vaginal vestibule in six subjects.^31^ Thermography was conducted after 10 min of vSculpt use (*n* = 2). This was compared with thermography after 10 min of use of a vibrator alone (*n* = 2) or after 10 min of use of the ApexM device (*n* = 2), which has an FDA clearance for treatment of SUI symptoms. The vaginal vestibule temperature did not rise more than 2°C above baseline when measured immediately posttreatment after vSculpt, vibrator, or ApexM use.

A second institutional review board (IRB)-approved study^32^ performed a temperature analysis to verify that the vSculpt device when used as indicated in human patients does not generate temperatures in excess of safe levels. Testing was conducted in 20 subjects to measure the temperature of the vSculpt device surface and the temperature of vaginal tissue during a 10-min treatment cycle. Pelvic examinations were performed by a board-certified OB/GYN pre- and posttreatment. No clinically observable changes were noted in participants' vaginal tissues posttreatment, nor were adverse events reported. The average baseline vaginal temperature was 37.3°C, and the average posttreatment vaginal tissue temperature was 37.9°C (*n* = 20). This represented an increase of 0.6°C over baseline on average (range, −0.6°C to 2.1°C). The maximum vaginal tissue temperature reached 39.2°C in a single patient. The average temperature at the surface of the vSculpt device after the 10-min treatment was 41.2°C (range, 38.6°C–44.1°C).

This study demonstrated that the surface temperature of the vSculpt and the vaginal tissue temperature maxima after a normal treatment session are within the 40°C–45°C range, which is typical of RF devices such as ThermiVa and is substantially lower than the local wound temperatures of 60°C–200°C that occur with CO_2_ and Er:YAG devices.^24–29^

The vSculpt contains nine LEDs placed circumferentially and disposed radially within a 2.5 cm section of the body of the device (Figs. 1 and 2). Six LEDs emit red light (662 ± 20 nm,15 mW, 125° viewing angle) and three LEDs emit NIR light (855 ± 30 nm, 15 mW, 130° viewing angle). The total power output of the vSculpt device is 1.4 W, with delivered energy densities of 12 J/cm^2^ at 6 min, 17 J/cm^2^ at 8 min, 22 J/cm^2^ at 10 min, and 24 J/cm^2^ at 12 min. The average power density is 34 mW/cm^2^. The location of the LEDs (Figs. 1 and 2) and their light distribution (Fig. 2C, D) are such that the photonic energy is delivered to the distal 5 cm of the vagina when the device is properly placed within the vaginal canal. The emitted light is directed radially and outward, rather than axially, along the shaft of the device during normal use and with normal depth of insertion of the device into the vaginal canal.

The depth of penetration of light within tissues is dependent on the incident wavelength, rather than the average power or intensity of the light source. However, a sufficient number of photons need to be applied to the tissue target to achieve an effect. The light distribution to tissue is affected by the design of the device as well as by the number and placement of optical sources within the device, the nature of the optical sources (i.e., laser, laser diode, LED, and SLD), and the distance between the device and the target or target surface. The incident beam from a laser source is generally coherent, collimated, and monochromatic (i.e., with a very narrow bandwidth). This contrasts with the light emitted from an LED source, which is neither coherent nor columnated, and these sources generally emit over a broader bandwidth, which is typically ±20–30 nm. LED sources also have a varying viewing angle, which further defines and influences the angle of divergence and attenuation of the emitted beam in both the forward and lateral directions.

The optical properties of the uterus, vagina, and adjacent structures are well studied.^27,29,33–38^ Bashkatov reported the depth of penetration of 660 nm light to be 1.6 mm and 850 nm light to be 2.25 mm in skin, with an associated absorption coefficient of ∼1.1/cm.^10^ The values for mucous membrane tissue were 3.5 and 5.8 mm, respectively.^33^ Madsen et al. studied the depth of penetration of 630 nm on the uterus, noting the depth of penetration was 2.59 ± 0.26 mm in postmenopausal and 4.79 ± 0.32 in premenopausal tissue.^36^ These values are consistent with the findings of Enwemeka who investigated the depth of penetration of 630 and 904 nm light on skin and muscle.^34^ Stolik studied a number of human tissues at several relevant wavelengths.^38^ The depth of penetration of light in the uterus was 2.40 ± 0.22 mm at 675 nm and 3.31 ± 0.02 mm at 835 nm; muscle values were 1.63 ± 0.10  and 3.72 ± 0.29 mm, respectively; and the depth of penetration of 675 nm light in the colon was 2.73 ± 0.29 mm.^38^ Hardy demonstrated that vaginal therapy at 1064 nm was better than urethral for treating SUI.^35^

Based on the depth of penetration data presented above and in consideration of the rapid divergence and attenuation of light emitted from an LED source as the distance from the source increases, it is reasonable to conclude that sufficient energy would be delivered to the distal vaginal and adjacent perineal structures, but that it is highly unlikely that the delivered light would be capable of reaching the uterus during normal use and with normal depth of insertion of the device into the vaginal canal.

The red and infrared light doses delivered by the vSculpt device are at irradiances used in various PBMT regimens. The rationale for treating the vaginal tissue with light energy is based on the hypothesis that the urethra will benefit from the PBMT effect, specifically by synthesis of collagen and elastin in the vaginal tissue and the supporting urethrovaginal sphincter muscle, and promoting vasodilation in the vaginal and urethral submucosa.

### Preliminary clinical data

An IRB-approved clinical study of vSculpt was conducted in the United States by de la Torre and Miller^94^ to measure the safety and effectiveness of the vSculpt on bladder and sexual function in women suffering from self-reported stress incontinence and sexual dysfunction issues. The protocol provided for enrollment of 55 patients at one clinical site. A sample size of 45 patients provided 90% statistical power to detect pre-to-post effect size ≥0.5 with a paired *t*-test and two-sided alpha = 0.05. A total of 55 women were enrolled in the study and 48 patients completed the study as per protocol.

Consecutive patients were evaluated for study eligibility by assessment of inclusion and exclusion criteria, medical history, and physical examination. Eligible patients were women, ages 30–59 years, with self-reported symptoms of SUI; postpartum with one or more vaginal births; painful intercourse with male partner; and/or dissatisfaction with intercourse. The main exclusion criteria were women who had an active sexually transmitted disease or infection; diabetes; neurological disorder; morbid obesity; current or attempted pregnancy; breastfeeding or lactating; history of cancer, chemotherapy, or radiation therapy; previous vaginal surgery or toning therapy; vesicoureteral reflux; bladder calculi or tumor; or conservative pelvic floor treatment (e.g., pelvic floor exercises and estrogen cream) in the last 6 months.

This study did not utilize a separate control group but compared patient outcomes with baseline. Given that the device emits a light that is visible and requires repeated home use, it was determined that a blinded control device (no light) would result in noncompliance treatment at home, thus invalidating the control. While patient-reported outcomes are susceptible to placebo effects, the 1-h pad weight test (PWT) is an objective standardized test that is less prone to such biases and can be evaluated against the patient's baseline. In addition, the magnitude of the treatment effects observed with urinary incontinence and sexual function questionnaire scores, as described in detail below, was greater than what might reasonably be expected due to placebo effect.

The primary efficacy end-point of the study using the FDA's Urinary Incontinence study guidance was the 1-h PWT, which is the objective measurement that most accurately measures SUI. Clinically validated QoL incontinence and sexual function questionnaires, including the Urogenital Distress Inventory-Short Form (UDI-6), the Incontinence Impact Questionnaire-Short Form (IIQ-7), the Female Sexual Function Index (FSFI), and Female Sexual Distress Scale-Revised 2005 (FSDS-R), and pelvic floor muscle strength (PFMS) using the standard Oxford Grading System were also measured as secondary efficacy end-points. Although this summary focuses on vSculpt's safety, a detailed discussion of the efficacy results was previously provided to FDA in Q170279.

The primary safety end-points were visual inspection of the vaginal tissue using the vaginal health index (VHI) scoring system by an OB/GYN practitioner and patient-reported incidents of adverse events, including discomfort with device insertion or use, local tissue warmth, nerve tingling, cramping, vaginal discharge, vaginal irritation, vaginal infection, or vaginal sensitivity. Voiding diaries were also used throughout the study.

The device was used according to its proposed treatment plan: every other day (3–4 × per week) for a duration of 45 days. The examinations were performed at day 1 and at approximately day 45, with the questionnaire being asked on days 1, 14, 30, and 45. Patients underwent 24 ± 5 treatments over 50 ± 11 days with the vSculpt device.

There were no serious adverse events reported among the women enrolled in the study. Vaginal inspection of the tissue by an OB/GYN practitioner using the VHI scoring system determined that there were no adverse changes to the vaginal tissue. Nonserious adverse events were reported by two patients (3.6%). One patient withdrew from the study due to self-reported excessive device warmth. One urinary tract infection was reported in a patient who did not properly clean the device after each use. The patient discontinued use for 2 weeks until the infection resolved, and then resumed therapy and completed the study with no further issues. No additional reports of adverse events, including discomfort with device insertion or use, local tissue warmth, nerve tingling, cramping, vaginal discharge, vaginal irritation, vaginal infection, or vaginal sensitivity, were observed at any time during the study.

The results of this study demonstrated that multi-modal vaginal toning therapy with the vSculpt device yielded clinically meaningful improvements in bladder symptoms, PFMS, and QoL in women with SUI. It is also noted that visual inspection in all study patients by the practitioner confirmed that there were no adverse vaginal tissue changes or events to the vaginal tissue.

Gaspar and Leibaschoff recently reported the results of a preliminary study of the effects of the use of the vSculpt and controlled exercises on the perineal floor of postmenopausal women.^95^ Two groups of eight postmenopausal women with symptoms of vaginal dryness were recruited. All patients previously underwent laser treatment for vaginal dryness with excellent results. The PBMT group received eight treatments with the vSculpt at a frequency of two sessions weekly in the clinic along with exercises guided by a perineometer over 1 month. The control group received the exercises alone guided by the perineometer twice weekly for a total of eight treatments over the course of 1 month. Subjective improvement was evaluated by visual analog scale (VAS) scores for dyspareunia.

Biopsies were performed in six patients. Baseline biopsies were taken in the left lateral vaginal wall at the junction of the middle and lower third using Tischler Morgan biopsy forceps. Posttreatment biopsies were taken in the right lateral vaginal wall at the junction of the middle and lower third (mirror image site) using Tischler Morgan biopsy forceps. Tissues were fixed with neutral formalin, embedded in paraffin, stained with hematoxylin and eosin, and evaluated by a pathologist using standard light microscopy.

**FIG. 3.**
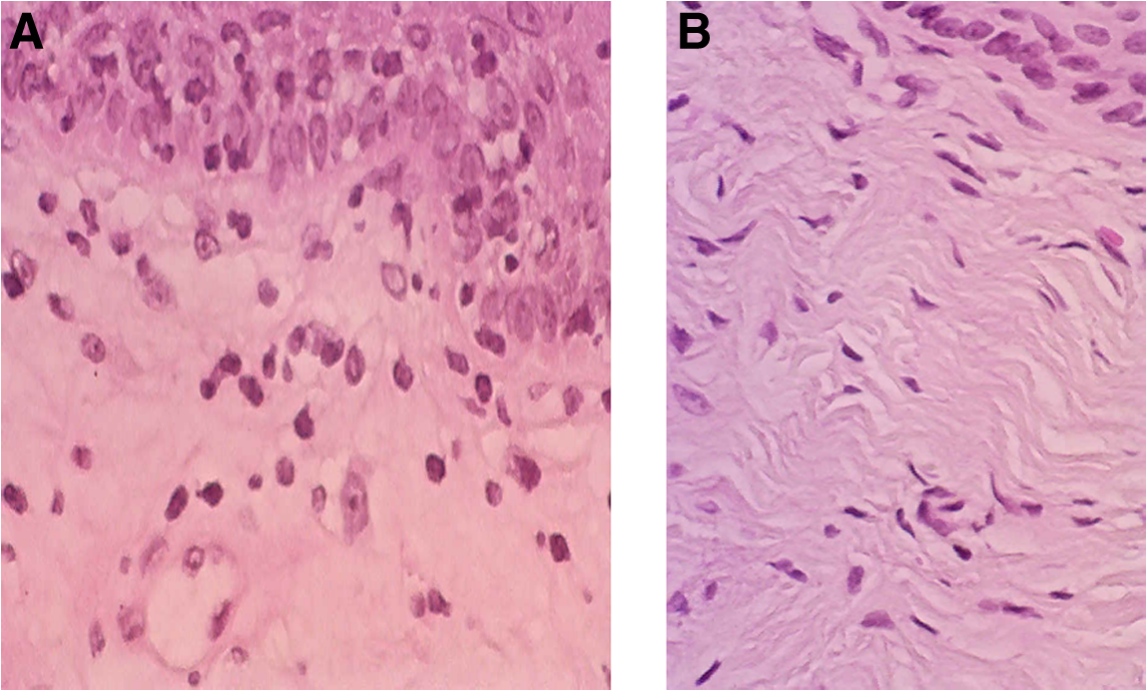
Histology from Subject 1 before **(A)** and 3 months after treatment **(B)** demonstrating restructuring of the lamina propria of the vaginal mucosa with collagenesis (100 × ).

**FIG. 4.**
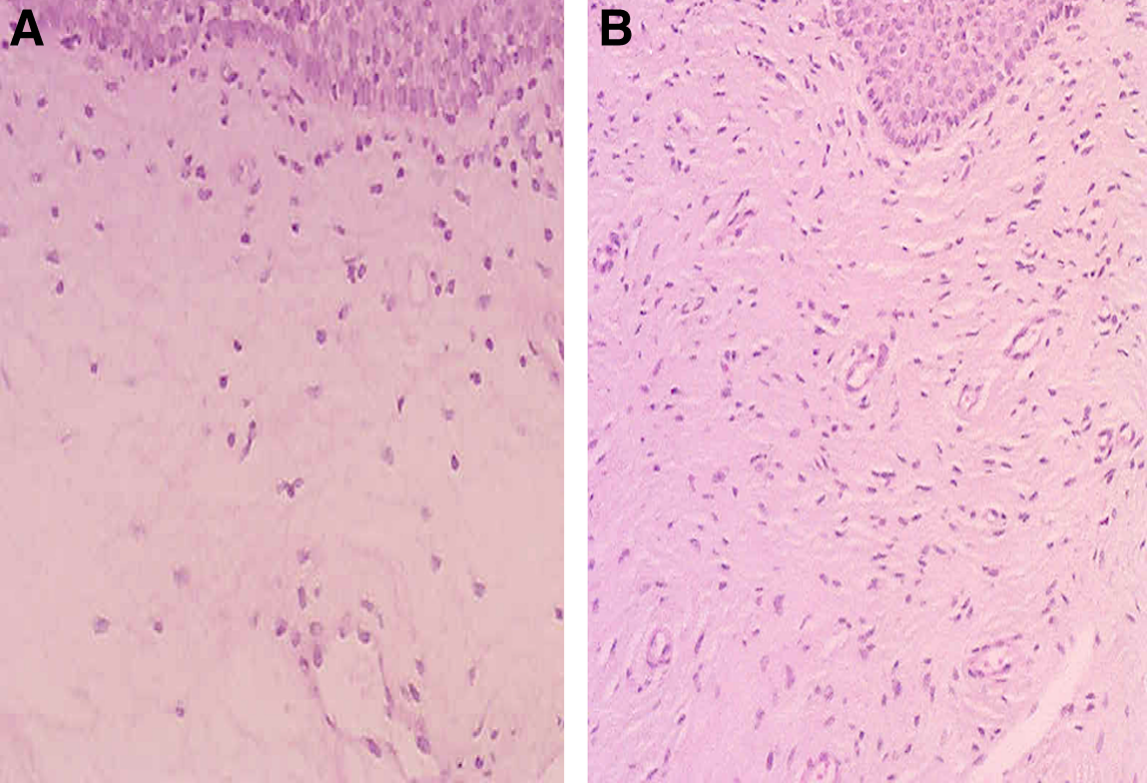
Histology from Subject 2 taken at baseline **(A)** and 3 months **(B)** demonstrating increased cellularity and vascularization (100 × ).

**FIG. 5.**
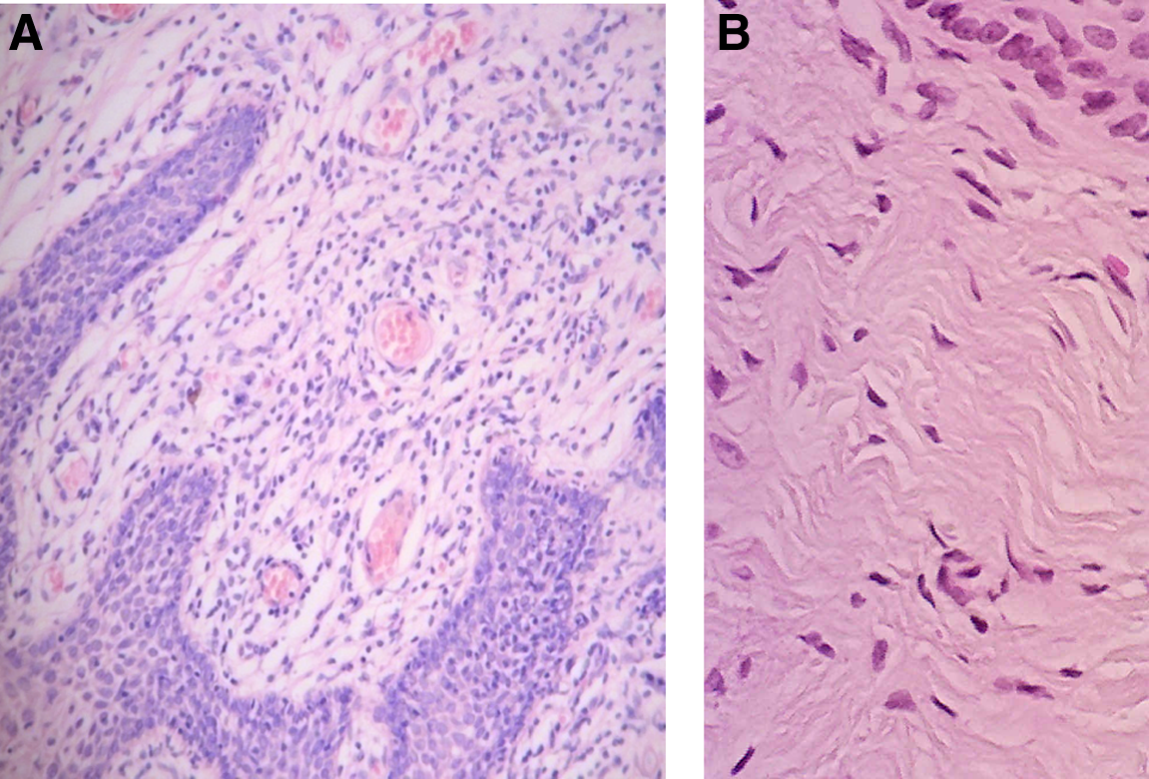
Representative histology demonstrating a complete restorative reaction at the level of the ECM with increased cellularity and angiogenesis 3 months after treatment **(A,** 40 × **)** and significant collagenesis **(B,** 100 × **)**. ECM, extracellular matrix.

**FIG. 6.**
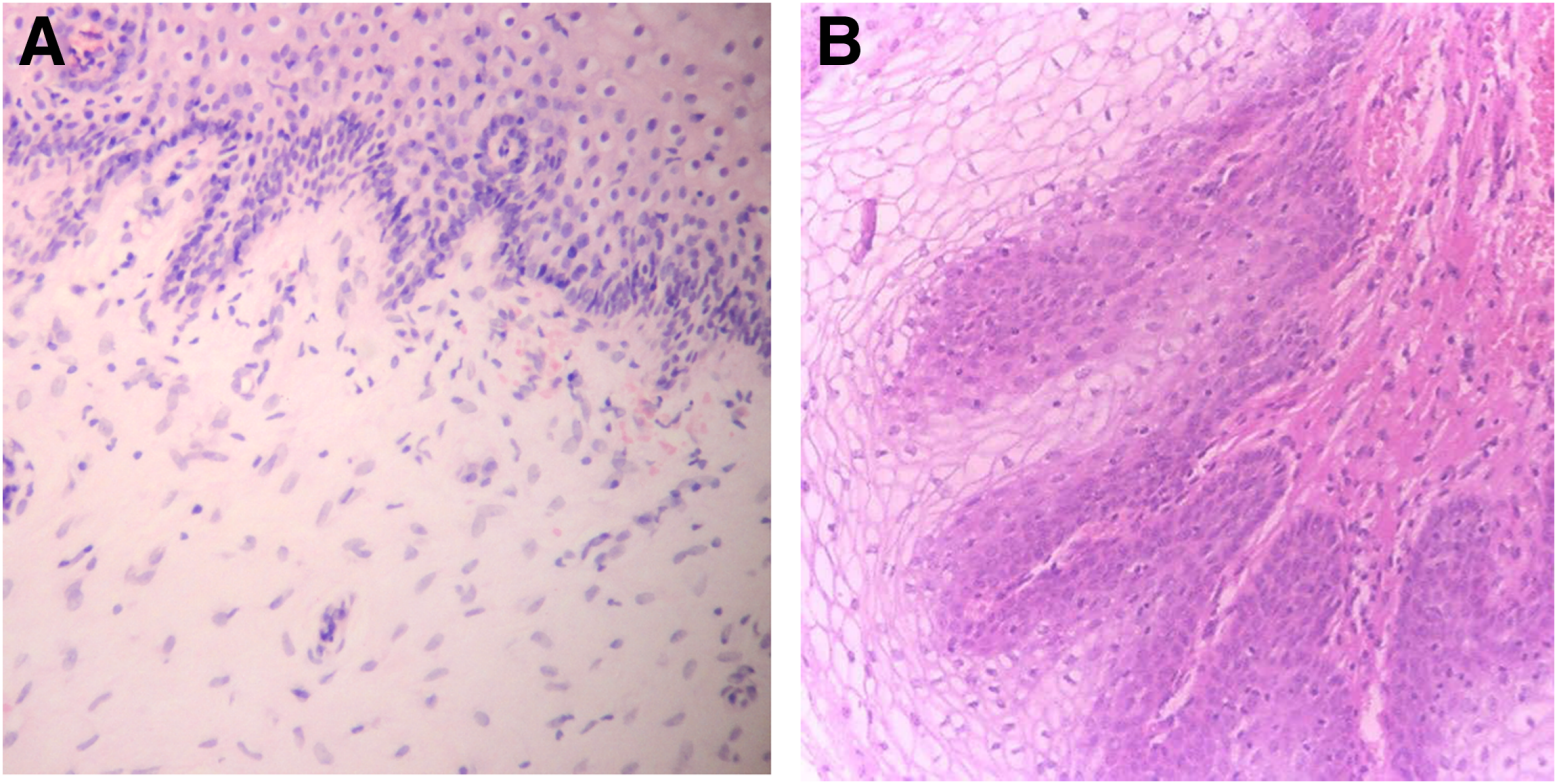
Histology of Subject 3 demonstrating papillomatosis, increased glucogenic load and thickness of the vaginal epithelium, with acanthosis and parakeratosis, increased cellularity, and a complete restorative reaction at the level of the ECM at 3 months after treatment **(B,** 100 × **)**. Compared with baseline **(A)**.

**FIG. 7.**
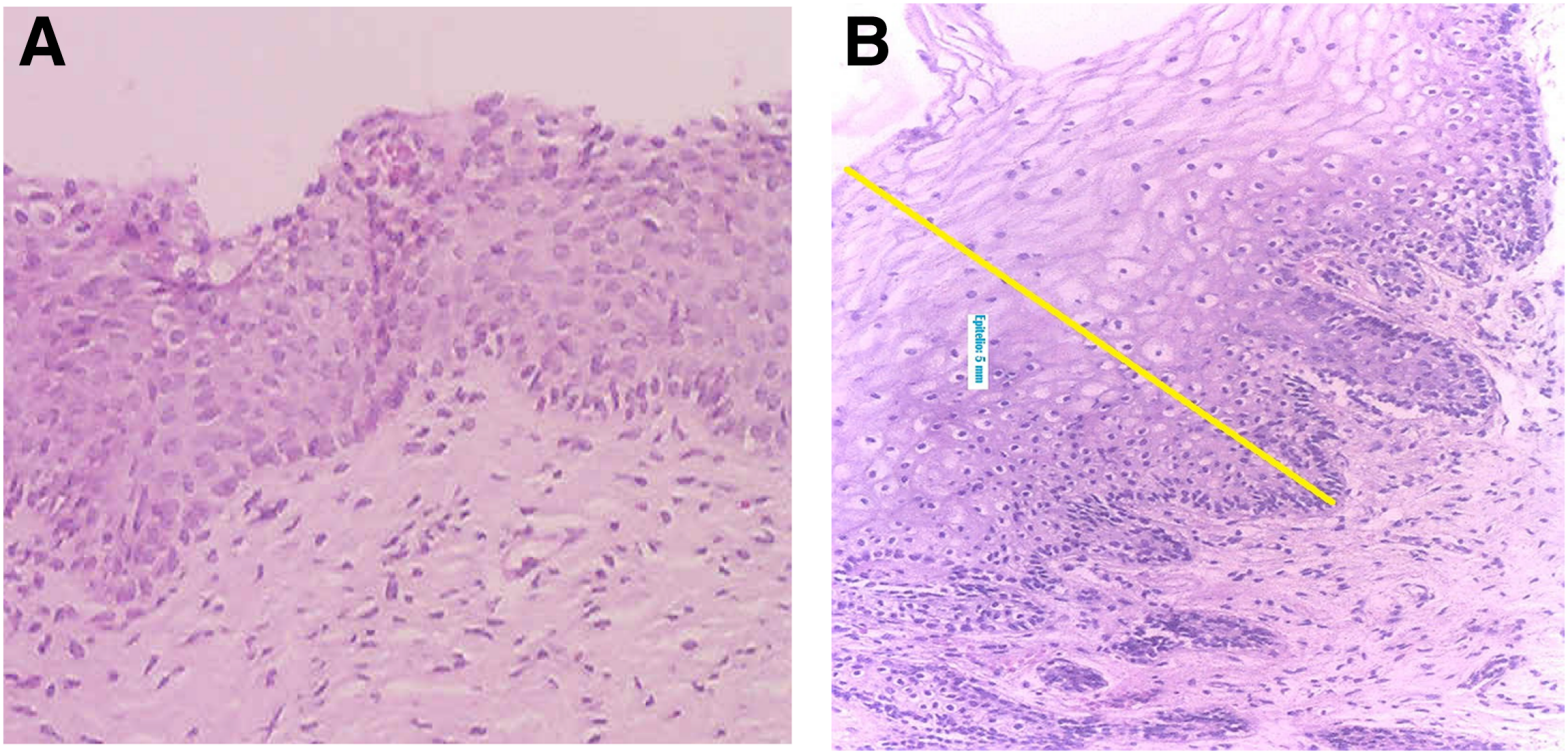
Histology of Subject 5 demonstrating noticeable changes at the level of the lamina propria from baseline **(A)** and at 3 months following treatment **(B)** with a 5-mm-thick epidermal layer (100 × ). The yellow line denotes marked expansion of stratified epidermis with a thick layer of glycogenated cells and elongation of the papillae.

**FIG. 8.**
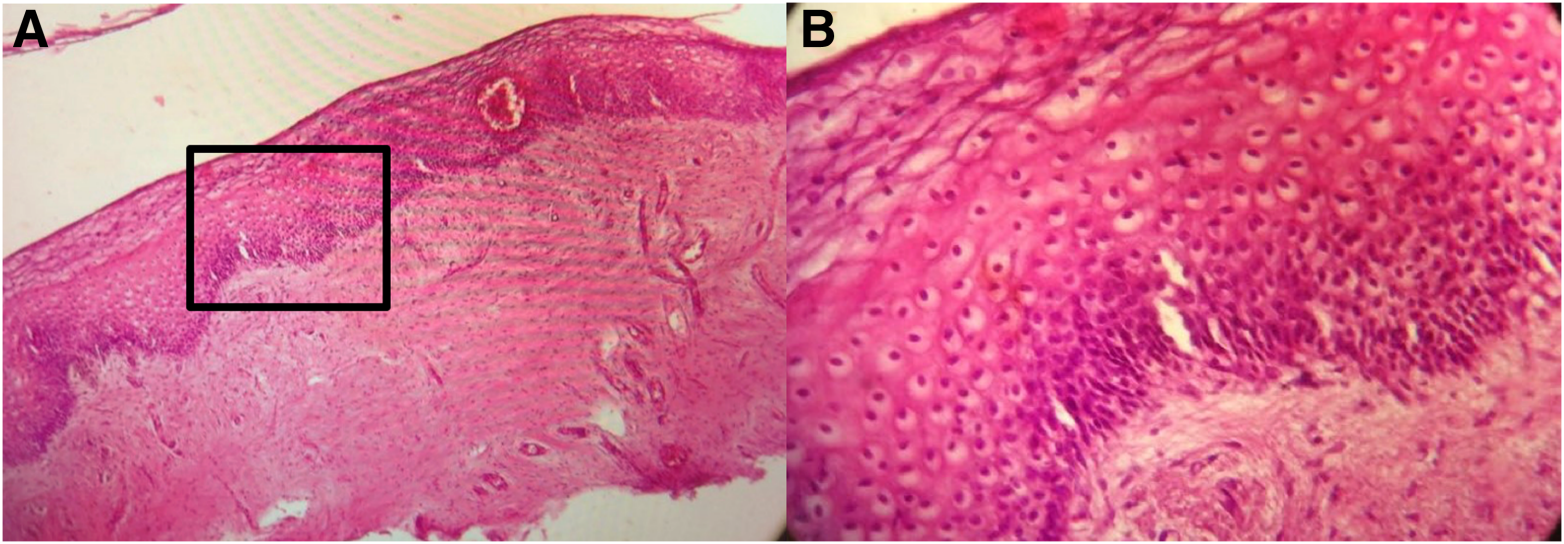
Representative histology in one study subject **(A** 40 × , **B** 100 × **)**. The inset in **(A)** corresponds to the area demonstrated in the higher magnification view in **(B)**. The vaginal mucosa is covered with a thick stratified glucogenic squamous epithelium with irregular acanthosis and papillae elongation. The basal layer is preserved and there is marked acanthosis and basal cell hyperplasia. There is mild parakeratosis and conservation of the superficial strata. The epithelium is nondysplastic and eutrophic. The chorion is edematous with foci of papillary hemorrhage, with capillary vessels and lymphatic ectasia. No signs of thermal injury are observed. Congestive vaginal mucosa is present.

Figures 3–8 demonstrate the results. The epithelial tissue consistently exhibited parakeratosis with an increase in keratinocytes, with acanthosis and an increased glucogenic load at 3 months posttreatment. Changes were also observed in the lamina propria at 3 months posttreatment, consisting of marked angiogenesis and evidence of congestion of tissues with red cells in new vessels, collagenesis, increased cellularity of the ECM, and papillomatosis. There was no clinical or histologic evidence of thermal injury or scarring during the course of the study or at the 3-month follow-up evaluation.

## Discussion

The vSculpt is designed for self-treatment at home based on the prescription of a physician. vSculpt uses the application of gentle heat and photonic and vibrational energy to the vagina, which are technologies already used in other medical devices and have a long history of safe use.^24–93^ The vSculpt device is designed to help stimulate tissue healing, reduce inflammation, restore cellular function, enable biosynthesis of new collagen fibers, and promote vasodilation thereby increasing circulation to the vaginal tissue, for improved vaginal health and temporary improvement of SUI symptoms.

Both the de la Torre and Gaspar studies^94,95^ demonstrate that the vSculpt device is capable of producing demonstrable and sustained clinical change in postmenopausal women with GSM and/or SUI symptoms. Further, the observed findings on clinical examination and histology are consistent with those observed with the already approved RF- and light-based technologies.^24–29^ The Gaspar study provides evidence that the subjective measures of decreased vaginal laxity posttreatment are supported by histologic evidence of collagenesis and normalization of tissues. There is no histologic evidence of dysplasia, metaplasia, or other abnormal changes in tissue after treatment. The Iguchi^28^ and Hardy studies^35^ in conjunction with the current understanding of the optical characteristics and absorption coefficients of the vagina, uterus, mucosa, and muscle^27,29,33–38,84^ and the design characteristics of the vSculpt provide evidence to conclude that the tissue effects of the vSculpt, like the other approved modalities, are limited to the lower genital tract and do not involve the uterus.

Pre-clinical and clinical data collected with the vSculpt confirm the intrinsic safety of the device.^31,32,94,95^ Specifically, the available information supports the use of red and NIR light delivered at parameters commonly used in PBMT regimens for improved vaginal health and the temporary improvement of SUI symptoms. It is understood that the current technology does not represent a pure application of PBMT as monotherapy, but is a combination therapy device that also incorporates the use of vibrational and thermal energy. Certainly, there is clinical evidence of the effects of thermal modalities on vaginal tissues. However, the current data demonstrate that PBMT could conceivably improve clinical outcomes via nonthermal mechanisms, and is a reasonable modality for application to the lower female genital tract. Additional studies using the current technology and trials using PBMT alone are warranted and should be encouraged.

## Summary

This article reviewed the biological basis, symptoms, and management of GSM and investigated the current status and rationale for the use of PBMT for improved vaginal health and the temporary improvement of SUI symptoms. Based on the preliminary evidence available, PBMT is safe and appears to be efficacious in treating GSM. These studies demonstrate that the vSculpt device is capable of producing demonstrable and sustained clinical change in postmenopausal women with GSM and/or SUI symptoms. The observed findings on clinical examination and histology are consistent with those observed with RF- and light-based technologies. While it is recognized that additional studies are warranted, PBMT represents a safe alternative in the management of these conditions.
